# Dr. James K. Donoghue

**Published:** 1915-04

**Authors:** 


### OBITUARY

Readers are requested to report promptly the death of all physicians in Western New York, or former residents of this region, or graduates of any medical school in Western New York, and to notify the families of the deceased of our desire to publish adequate obituary notices.

**Dr. James K. Donoghue,** Georgetown (D. C.) 1909, formerly a member of the staff of Central Islip Hospital, Long Island, but for the last year a practitioner of Rochester, died in Rochester, February 14, aged 28. Death was due to pneumonia.

